# A vibrant reflection of the revised integrated school health policy with a lens on substance use

**DOI:** 10.4102/phcfm.v13i1.3082

**Published:** 2021-10-28

**Authors:** Linda Shuro, Firdouza Waggie

**Affiliations:** 1 School of Public Health, Faculty of Community and Health Sciences, University of the Western Cape, Cape Town, South Africa; 2 Department of Clinical and Community Engagement, Faculty of Community and Health Sciences, University of the Western Cape, Cape Town, South Africa

**Keywords:** school health policy, substance use, South Africa, adolescents, integrated political approach, learners

## Abstract

Substance use is rife amongst adolescents, including learners. Learners are easily exposed to substances with onset as early as 10 years and average age of drug experimentation is 12 years in South Africa. This results in many negative health and social outcomes, a challenge as far as the achievement of global, regional and national goals such as quality education. The revised Integrated School Health Policy (ISHP) is a policy operating within the school environment aiming to address health and social barriers of learners and improve optimal health, comprising a vague action component on substance use prevention. This article is an opinion piece, which uses the Walt and Gilson model as an operational framework to analyse the revised ISHP within the lens of substance use. It assesses the four interrelated aspects: policy context, policy content, policy actors, and the policy process. The ISHP is placed within schools where adolescents are found and has the potential to reduce many health challenges such as substance use amongst learners. However, some issues are left to chance, such as health education on substance use prevention stated to only begin at Grade 4 (10 years), little mention of parental involvement, limited interplay amongst actors, limited investment in upskilling educators on dealing with substance use, scarce resources for implementation in all developmental phases and provinces to address substance use. Intervention can be more comprehensive with an intersectoral political approach needed to ensure that implementation addresses all multiple levels of influence of substance use amongst learners and the numerous health and social barriers.

## Introduction

Substance use amongst adolescents in South Africa (SA) (majority of them are in school) is an escalating public health concern, with an early age of onset (10 years)^1^ and a mean age of drug experimentation at 12 years.^2^ Adolescents are exposed to substances such as alcohol, cigarettes and illicit drugs.^3,4^ Marijuana (dagga/cannabis), nyaope (mixture of heroin and other low-grade substances) and crystal meth (also known as TIK or CIK) are some of the illicit drugs with far-reaching effects on individuals, families, communities, educational and health outcomes.^3^ A quick glance at trends in drug use over the years amongst learners, shows that prevalence rates have remained unchanged and sometimes increased in some provinces in SA.^5,6^ The 1st, 2nd and 3rd Youth Risk Behaviour surveys conducted in SA show that in 2002, 2008 and 2011, the percentage of learners who had ever smoked cigarettes were 30.5% (one in three), 29.5% and 28.0%, respectively. In terms of alcohol consumption in a lifetime, the percentage of learners was 49.1% (2002), 49.6% (2008) and 49.0% (2011). Marijuana rates in a lifetime were 12.8% in 2002, 12.7% in 2008 and 13.0% in 2011.^7,8,9^ Over this time period, the age of first use is still reported to be under the age of 13 and the given statistics show little or no significant reduction in use.^10^ Alcohol, cigarettes and cannabis use prevalence still remains high in the age range 15 and older, in which adolescents fall. This gives an indication that prevalence rates are not significantly decreasing despite existing public health interventions targeted at adolescents. It raises a concern because of the negative effects associated with substance use, with a high chance (60%) of learners repeating a grade and truancy.^3^

Simultaneously, young people’s access to health promotion initiatives varies widely during formative years.^11^ In SA, there are many public health interventions developed to address health issues amongst adolescents. Policies within the school environment provide a course of action to address many of the educational, health and social challenges.^3,4,12^ Considering these many policies relating to children and adolescents in SA,^13,14,15,16,17,18^ it is worrying to note a gradual increase in prevalence rates of substance use amongst learners, questioning, what are the possible gaps? One such policy is the revised Integrated School Health Policy (ISHP), a public health policy, launched in 2012 as a collaboration between the Department of Basic Education (DBE) and Department of Health (DOH).^19^ Prior to 1994, SA had a historical past of apartheid with segregation and inequalities. Post-apartheid the new administration embarked on a process to reduce these differences and bring improved quality to people including school health services; hence, the policy is a result of this transformational change for equality.^20^

This article is a brief analysis of the revised ISHP in SA with a lens on substance use.

## Methodology

The comprehensive *Walt and Gilson model* is used as the operational framework for a perspective analysis of the revised ISHP, as it incorporates a political analysis of health policy.^21^ It recommends analytical attention to four interrelated aspects of the policy: *policy context, policy content, policy actors and policy process.*^22,23^ Analysis of the policy context looked at some of the contextual factors (history, concerns, ideas, etc.) at play and what ways do these influence the ISHP specifically on substance use prevention. The full range of policy actors was identified and how they influenced the policy. The past school health policies and how the current content of the ISHP influences curb substance use in schools were mapped. The policy processes (chronology of steps) deployed were stated reflecting the process of change.^24^ The model is deemed reliable and has been used in several studies to analyse policy especially in low- and middle-income countries.^25^

The analysis also draws on the University of Western Cape (UWC) School of Public Health (SOPH) *module guide*, which provides a complementary methodological approach to analyse each of the aforementioned four categories of the policy. The approach (in a table format) breaks down the category into issues raised and what the issue links to or influences over other issues.^24^ The analysis is also guided by the *Health Policy Analysis* (*HPA*) reader which provides features critical to include in each of the four aspects of the policy analysis, as well as examples of selected papers that have applied the Walt and Gilson model.^25^

### Analysis of the revised integrated school health policy

Table 1^24^ gives a summary analysis of the policy context, content and actors of the ISHP and the following selection provides a brief narrative reflection of the four interrelated aspects of the policy.

**TABLE 1:** A summary analysis of the policy context, policy content and actors of the revised integrated school health policy.

Category of issue	Issues raised	Issue links to/influences over other issues
Context	South African government committed to put children first	Signatory to the convention on the rights of the child
	Policy framed within a global, regional and national context	Adapted many regional instruments and embedded within the framework of relevant policies: Millennium development goals now Sustainable Development goals, 2000 EFA Dakar Framework for Action to achieve Education for All, Care and Support for Teaching and Learning Program, The Health Promoting Schools Programs (HPSP)
	Most children are in rural areas and live under the income poverty line; depend on social grants; child headed households; access to basic services limited; oral health-tooth decay; HIV and AIDS; mental health; substance use; crime; trauma and violence^5^	The context still remains the same.
		Substance use increased use and prevalence of dagga use very high^7^ with increased use in earlier ages.
		Substance use is still a priority risk behaviour amongst learners.
	Structures proposed from a national to school level (national, provincial, district, facility and school-based task teams)	There is limited intersectoral implementation
Content	Vision, goal, objectives, target group and those not covered by the school health programme	Vision and target group the same as from the previous policy
		Goal phrased differently but has not significantly changed from the previous policy except for collaboration
	School health package of services: Health education and promotion. Learner assessment and screening The school health package (foundation, intermediate and senior phase) Provision of onsite services Follow-up and referral Coordination and partnership Community participation; learner participation Consent and assent	Limited resources affecting implementation^1^
		The mention of drug and substance use comes as a sub-item under mental health on the health education and health promotion. It is one of the topics to be covered under life orientation and co-curricular activities. As a result of an increased prevalence, it should be itemised as a bullet on its own to receive the much-needed attention as with other priority issues.
		Under school health package services, substance use is mentioned under health education starting from intermediate phase (Grade 4–6) and going on to senior phase (Grade 7–9) and FET phase (Grade 10–12). This is a missed opportunity for early health education to prevent experimentation and initial use as statistics indicate early onset of substance use by learners.
	Implementation guidelines and structures at national, provincial, district, PHC facility and school level	Resource package for School health nurse available
	Mention of the monitoring and evaluation plan	-
Actors	Ministry of Health and Ministry of Education.	Policymakers and key actors in implementation
	School health nurse -implementers at the schools with collaborators	Shortage of nursing staff in the health system
	Department of Social Development	Key collaborators
	Learners from Grade R to Grade 12	Target group/Beneficiaries children not covered by the programme
	Non-governmental organisations (NGOs) such as UNICEF^1^, UNFPA^1^,	Collaborators:assistance in finalisation of the policy, resource package for the school nurse and appraisal of the previous policy
	Section 27, Equal Education	Pressure groups

*Source*: Lehmann U. Master’s in public health understanding and analyzing health policy. Cape Town: School of Public Health University of Western Cape; 2016

EFA, Education for all; HIV, human immunodeficiency virus; AIDS, acquired immunodeficiency syndrome; PHC, primary health care; UNICEF, United Nations Children’s Fund; UNFPA, United Nations Population Fund; FET, Further Education Training; NGO, non-governmental organisation.

#### Policy context

A myriad of challenges exists in the school environment including the persistent rise of substance use by learners. The policy context seems favourable to address the health and social barriers faced by learners because many legislative frameworks are aligned and incorporated within the revised ISHP.^19^ Approximately one in every five adults in SA are engaged in substance use. Alcohol, tobacco and dagga are commonly used with onset likely to have begun in adolescents.^3^ The rate of increase in illicit drug use in SA is sometimes described as a lost battle.^26^ This has an implication and cost to the country because of the increased absenteeism at work and school, reduced productivity, health costs and crime as some of the examples. Cannabis is the most commonly used substance in Western Cape, Northern Region (Gauteng province, Mpumalanga and Limpopo provinces) and there was a significant increase in alcohol-related cases of patients under 20 in KwaZulu-Natal.^27^ The most commonly abused substances amongst adolescents are dagga, alcohol and cigarettes. The prevalence of heavy episodic drinking in 2016 amongst 15- to 19-year-old was 20.3% in males and 4.0% in females in the past 30 days and 12.8% as average in both sexes.^28^ The average age of drug experimentation in SA is 12 years and this is dropping.^29^

The revised ISHP was introduced as a framework for the new school health programme, implemented at sub-district level in 2012. The policy came in after a historical background of segregation and inequalities pre-2000. Post-apartheid led to the development of the 2003 National School Health Policy to redress the historical background of inequalities in school health. School health did not improve much and some failures were attributed to a lack of collaboration and the policy reduced to a DOH initiative despite initial efforts by many actors, which included researchers. The ISHP is a revised initiative located within national, regional and global frameworks to promote education and health^15^ and includes a health education component on substance use prevention. It incorporated pro-collaboration health reforms such as re-engineering primary healthcare, which aligns with and includes school health services. The 2010 State of the Nation address gave political leverage for the reinstatement of school health services and influenced the launch of the ISHP in 2012.

Although the ISHP outlines the role of respective departments in addressing the health needs of learners to ensure a strong school health service, literature suggests that there is stagnancy in implementation and working in silos of sectors.^30^ An integrated political approach aligned with the numerous policies is needed to address substance use and other challenges in the school environment.^20,30^

#### Policy content

The analysis of the following policy characteristics of the ISHP helps to get an understanding of the policy goal and objectives in the prevention of substance use by learners:

- *Cost and benefits*: The policy intends for learner barriers to be addressed for optimal health and better educational outcomes. However, the differentiated implementation creates scope to exacerbate inequalities in terms of access to services with some schools and provinces getting more support than others. Even in the proposed quintiles one and two schools’ where activities should have started, implementation is still very slow.^30^
- *Administrative/technical content*: The ISHP incorporated substance use prevention, as part of the education curriculum. The Life Orientation subject is intended to cover health education on mental health issues including substance use, depression, anxiety, suicide and is supplemented through co-curricular activities. Education on anti-substance use starts from Grade 4, which seems delayed considering that the age of onset is decreasing. Age-appropriate early education starting with the foundation phase and an integrated approach to substance use education is needed in all subjects. The ISHP promotes the need for teams to develop an ISHP monitoring and evaluation plan but there is no guidance in the policy on the development of such a plan. However, an evaluation of the National Health Insurance (NHI) phase 1 pilot interventions, which included the ISHP observed the importance of the policy but the lack of clarity on roles, coordination, integration and human resource constraints, coverage problems with limited or no visits by school health teams.^30^ These ambiguities and continued problems have an impact on any intervention and may be affecting any efforts to curb or address substance use amongst learners.
- *Extent of participation:* There is no explicit mention of parental involvement in the school health services, except for the consent of learner participation in health and wellness programmes. Parents play an important role in nurturing information obtained beyond the school environment. This is a major gap in the ISHP policy, as parental involvement is crucial in anti-substance use initiatives.
- *Resources:* A study reviewing school health nurses’ roles and responsibilities regarding prevention of drug abuse by learners indicated that the allocation of professional nurses is not proportional to the magnitude of the substance use problem.^31^ In some provinces, the DBE was placed under administration affecting implementation and availability of resources in schools.^32^ Without explicit commitment to funding for the ISHP, mental health, for instance, which also addresses factors leading to substance use will consequently be neglected.

#### Policy actors

The South African president recognised the school health programme during the 2010 nation address leading to the launch of the revised ISHP in 2012.^19^ This set a foreground for collaboration possibly bringing more actors to be involved in school health programmes and the drive for collaboration than the previous school policies.^33^ The main beneficiaries of the policy are learners from Grade R to Grade 12. The primary drivers of ISHP are the DBE and DOH. Department of Social Development (DSD) is a main collaborator addressing social issues. As a result of the differences in the implementation of the policy in the various provinces, pressure groups and NGOs push for change to collaboratively address these policy implementation challenges. These include Section 27, Equal Education, Treatment Action Campaign, UNICEF, UNFPA, SPW SA Trust, South African National Council on Alcoholism and Drug Dependence (SANCA) and Love Life.

The school health nurse from the nearest health facility, together with a team plays a key role in the implementation of the school health programme but is not always available owing to the shortage of health workers. Another important actor that should be on the forefront to receive training is the parent or guardian, barely mentioned in the policy and often, only involved when the problem has escalated. If parents are empowered regularly, they can detect and solve a lot of issues at family level. Educators are found at the frontline of dealing with learners abusing substances and are often not adequately equipped to respond effectively to the challenges such as early detection of substance use. Similar to a resource package for the school health nurses,^34^ there should be a resource package for all educators on issues such as testing and how to respond to intoxicated learners. It is important to upskill the different actors so that they can execute their roles. Most actors, for instance, school managers are aware of the ISHP but have not received adequate skills training for implementation.^35^ The presence of school based social workers, regular visits by counsellors and/or psychologists working with the educators is needed and will help to assess factors that end up affecting learners psychologically and result in resorting to substance use. Social workers are not placed in all schools suggesting an urgency for a memorandum of understanding (MOU) between DSD and DBE to incorporate this post permanently within schools. The Department of Social Development role should be more imminent as they play a critical role in financial support for substance use prevention programmes, alleviating poverty and addressing some of the factors that result in substance use amongst learners. The department’s policies such as the *Integrated Plan on Early Childhood Development* included in the ISHP can kick-start age-appropriate substance use prevention programmes at an early age as part of preventive and promotive services.^19^

From an ecological perspective, substance use amongst learners cannot only be addressed through mental health assessment and health education because of the multiple levels of influence. A study describing compliance of ISHP in terms of collaboration in Tshwane highlighted ‘insufficient stakeholder integration’ in the implementation of the policy.^36^ Tackling of health and social barriers faced by learners requires an interplay of sectors and their presence in the proposed ISHP structures (national, provincial, district, facility, school and community). The SANCA and South African Police Services (SAPS) are some of the key actors that should be engaged more in prevention initiatives and as such an MOU between the DBE and SAPS^37^ exists. Certain schools (in Limpopo) have fostered such relationships to address substance use. The legal age for the sale of alcohol is at 18 years, yet learners as young as 10 years old are abusing substances. The assumptions are that an ‘illegal market’ is thriving. It is crucial that sectors of Justice and Trade impose stricter/tighter measures to support any efforts of the ISHP in addressing substance use.

#### Policy process

A chronological order of the process of change of the ISHP is summarised in Table 2.^24^

**TABLE 2:** A summary analysis of the process of change of the revised integrated school health policy.

Category of issue	Issues raised	Issue links to/influences over other issues
Processes of policy change^1^	2003: National School health policy (NSHP) launched	A means to rectify the differentiated school health services emanating from the apartheid era, poor reach, under resourced, poor collaboration.
	2009: Health and education policy reforms which included school health	The policy is embedded in the health sectors’ response to strengthen through re-engineering PHC and the education sector through the CSTL programme.
	2010: A directive from the president to reinstate school health programme through the 2010 Nation Address	More actors were involved in assisting with finalising the policy and resource package for the school health nurse: UNFPA, UNICEF.
	2012: The ISHP launched as revision from the 2003 NSHP and revised from the 2011 NSHP	A shift to planned better collaboration between the DOE and DOH and DSD. Policy co-signed by both DOE and DOH. Department of health provides services and DOE creates enabling environment.
	2020	Policy implementation still has insufficient integrated intervention; skills and expertise required for implementation.

*Source:* Lehmann U. Master’s in public health understanding and analyzing health policy. Cape Town: School of Public Health University of Western Cape; 2016.

PHC, primary health care; UNICEF, United Nations Children’s Fund; UNFPA, United Nations Population Fund; DOE, Department of Education; DOH, Department of Health; ISHP, integrated school health policy; CSTL, Care and Support for Teaching Learning; DSD, Department of Social Development.

A quick comparison of the 2003 NSHP and 2012 ISHP highlights the improved scope of the inclusion of substance use prevention allocated under the health education and health promotion component, with health education to begin from intermediate phase (Grade 4–6) up to FET phase (Grade 10–12). The 2012 policy specifies for the health education to be part of Life Orientation and co-curricular activities. However, prevalence rates of substance use remain unchanged and, in some provinces, increasing amongst learners. There is a need for more detailed content for upskilling players on the prevention substance use. Simultaneously flexible collaborative activities for buy in of implementers and fostering collaboration with other role players in addressing substance use should be developed. The ISHP is potentially placed to address the determinants of health and development within school children, as it incorporates progressive health reforms such as comprehensive PHC and the Care and Support for Teaching Learning (CSTL) framework.^16^ Whilst there is a strong service delivery component (largely screening),^31^ it is important that there is an explicit intersectoral approach in the development of activities and implementation of the policy and the use of existing approaches such as HPS, which help to create a supportive environment for improved health and educational outcomes.^38^

### Challenges in implementation

- Several departments were placed under central government administration including health and social development affecting resource availability^32,39^ and implementation of any policy including the ISHP.
- There were disparities in the implementation of the ISHP as not every school has school health nurses visiting and school health services implemented in certain phases, which means that not all students are screened and referred timeously. It may mean late intervention for learners abusing substances.^30,31^
- Inadequate training for school managers and educators who are unaware or have a vague understanding of the ISHP has implications for coordinated activities to reduce substance use.^36^
- Intersectoral collaboration is still very slow with a lot of sectors working on substance use prevention in silo programmes.^30^

### Implications and recommendations

The following points are for consideration:

- An integrated political approach is needed to address substance use amongst learners with different sectors, with the revised ISHP broadening as a joint initiative that includes more sectors such as police, justice, social development to ensure that implementation addresses the multiple levels of influence onset of substance use.
- There is a need for expertise such as counselling and school-based social workers to timely identify mental and social problems, which may result in substance use. These should work with the school health nurse and ensure timely referral and intervention.
- Regular training of educators and parents on anti-substance use prevention approaches and a significant component of parental involvement on policy implementation including substance use to operationalise it at school and community level.

## Conclusion

The revised ISHP is grounded in the school environment with great potential to address many health and social challenges of learners. Intersectoral collaboration for the implementation of the ISHP with commitment and resources is imperative in order to address the multiple levels of influence of substance use amongst learners. Substance use is an increasing epidemic causing havoc in learner’s lives. If existing policies and sectors interplay effectively with the ISHP, substance use can be curbed amongst adolescents.
